# Food systems transformation: rationale and methodologies

**DOI:** 10.1098/rstb.2024.0146

**Published:** 2025-09-18

**Authors:** Peter Horton, Margaret Gill, Guy Poppy

**Affiliations:** ^1^ School of Biosciences, The University of Sheffield, Sheffield, UK; ^2^ School of Biological Sciences, University of Aberdeen, Aberdeen, UK; ^3^ School of Biological Sciences, University of Bristol, Bristol, UK

**Keywords:** food, environment, agriculture, nutrition, health

How to transform the failing global food system to provide a resilient, sustainable food future is one of the most urgent problems facing humankind [1]. Global changes in food demand and dietary habits are placing an unsustainable burden on healthcare systems and environmental sustainability at the same time as several emerging threats to the production and supply of food. There is no shortage of reports and academic papers making the case for the need to transform food systems, many of which are cited in this Theme Issue. A wide spectrum of evidence documents the long-term unsustainability of how we produce food, how it is processed and the negative health consequences of our diets. This includes: data on child malnutrition and the consequences for brain development; the negative impact of diet-related disease on labour productivity and premature deaths; the destruction of natural resources required to support food production; the high contributions to greenhouse gas emissions; and predictions of the increasing difficulty of maintaining food supplies at the scale to feed the world’s population amidst socioeconomic, political and environmental challenges. The evidence of the need for change is clear, but several factors make it very difficult for governments to identify effective policies. These include: the size of the contribution of the food sector to the economy (for the UK in 2022 this was £147 billion or 6.5% of economic activity as gross value added and 4.2 million or 13.4% of employment [2]); the impact on rural life; the complexity of interactions globally; and the power imbalance between producers and retailers.

Traditionally, agriculture and nutrition were seen as the disciplines most relevant to feeding the world but now the list is longer. It extends from genetic engineering to process engineering involving ways of blending foods from multiple constituents, and from simple economics of supply and demand to economic modelling to drive the sourcing of foods geographically to ensure year-round supply. Therefore, in recent years, there has been a move to interdisciplinarity (research questions in one discipline being informed by linked research in another discipline) and trans-disciplinarity where sectors beyond research (in this case various players in the wider food system) are engaged in co-design and co-delivery of research projects (discrete pieces of research) and programmes (interlinked projects). It is within this context that United Kingdom Research and Innovation (UKRI) invested £47.5 million into a programme ‘Transforming the UK Food System for Healthy People and a Healthy Environment’ (TUKFS). The objective of the programme was to fundamentally transform the UK food system by placing healthy people and a healthy natural environment at its centre [3]. Led by one of us (G.P.), it involved almost 40 research institutions and more than 200 organizations in the private and third sectors as well as more than 10 UK government departments and agencies. The research addresses questions around what we should eat, produce and manufacture. Crucially, it considered the complex interactions between health, environment and socioeconomic factors, exploring the potential trade-off involved to ensure that solutions to problems in one area do not deliver at the expense of another. Thus, interventions to increase availability and affordability of healthy foods also need to ensure profitability and viability of the farmers and companies involved. Similarly, measures to increase environmental quality cannot be introduced at the expense of food security (and vice versa). While there are repeated calls to address the food system at a global level, the focus of the TUKFS programme was on actions at the more national level which are more likely to produce excellent research that has impact [4].

This Theme Issue describes some of the major outputs from the TUKFS programme: new data, evidence and conclusions covering key components and stakeholders of the food system. Its novelty and distinctiveness rest upon the interdisciplinary nature of the research, combining science, technology, social science and public health. It presents an integrated view of the food system, starting from a perspective of what is required for healthy humans and minimizing the environmental impacts arising from food production. It includes several case studies, focusing on the urban food system where inequality in access to food represents a key health risk. An international dimension is included to explore the transformations in food systems taking place in other countries, with food systems both similar to the UK and divergent from the UK.

The first challenge in successfully transforming the food system is to develop the novel approaches that enable the required interdisciplinary research. Social science is at the centre of this challenge. The paper by Lyon *et al.* [5] introduces the concept of social innovation to better understand how to bring about social change and innovation in how people grow, trade and consume food. Sawyer *et al.* [6] explore this issue further, focusing on how to overcome the intransigence to change within the food system, offering a case study of how interdisciplinary science, systems thinking and stakeholder engagement can identify transformative interventions. The array of social factors that determine human health introduces an additional complexity in this process. The paper by Wagstaff *et al.* [7] describes the impact of these social determinants, such as housing, employment, education, power and discrimination, together with psycho-social factors such as isolation, social networks and perceived level of control into food system transformation. Integration of the various factors and processes involved in the food system, and establishing the potential trade-off involved, requires a quantitative methodology to combine the assessment of the nutritional benefits and environmental impact of food. Grigoriadis *et al.* [8] describe the Sus-Health Index, which is a product of existing nutrition and environmental indices co-created with a range of stakeholders in policy, industry and consumers. The important benefit of such metrics is that they can help inform consumer choice.

It has been established that changing diets is essential not only for improving health but also for reducing environmental impact; but, how can dietary change be catalysed? As emphasized above, social interventions are central to this. Lovegrove *et al.* [9] discuss the strategies that could be developed to increase the intake of dietary fibre, proved to have health benefits, including better nutritional education, public health messaging, more informative and effective food labelling and food reformulation. One of these strategies is explored by Wilkinson *et al.* [10], the promotion of dietary change in school food. They found that fibre intake could be readily improved by replacing white bread with high fibre bread in school breakfasts and suggest that simple policy levers in schools could be effective in generational dietary change. The paper by Flynn *et al.* [11] proposes an entirely different approach to inducing dietary change in a canteen setting with weekly menus. The idea is to change the competition structure between menu options and thereby influence the frequency with which more sustainable options are selected. No dish is withdrawn, no recipe reformulation is required and diners remain unaware of any intervention. Applying computational mathematics to analyse menus, this approach was modelled using menus from 12 NHS hospitals across the UK, combined with responses from an online food choice task undertaken by 50 participants per region. It was predicted that this approach could result in a significant reduction (6–31%) in either the carbon footprint or saturated fatty acid intake.

Dietary change is also linked to food production: what food is grown, how it is grown, where it is grown and how it is processed? Ingram *et al.* [12] take a radical approach, introducing the idea of reverse engineering a food system: rather than *farm to fork*, they describe a *fork to farm* approach to promoting healthier diets with lower environmental impact. They introduced dry bean varieties grown in the UK into institutional catering and home cooking, and assessed the overall effects on health, food sourcing, farming and the environment together with local and national economic benefits. An equally radical approach is described in the paper by Kurhan *et al.* [13] which describes how edible nutrients can be extracted directly from grass, serving as the raw material for protein-rich healthy food while by-passing the need to use livestock. This would help to mitigate the greenhouse gas emissions associated with ruminant production and increase resource-use efficiency.

As with all new approaches in the food system, the social implications of the interventions in food production need to be fully understood. The final two articles from the TUKFS programme explore these implications in relation to other innovations in food production. Kluczkovski *et al.* [14] focus on vertical farming, widely promoted as offering fresh healthy food while reducing the environmental impact and risk of arable farming and so contributing to more sustainable and resilient food production. This paper explores the indirect social benefits of vertical farming in terms of greater equality of access to food, creation of green jobs, community development and civic participation, and what policy measures are needed to make these happen. Regenerative agriculture has similarly been put forward as the principal way in which the environmental impact of farming can be reduced, with soil quality and biodiversity enhanced. Berthon *et al.* [15] describe research aimed at providing evidence of the environmental and socioeconomic outcomes of specific regenerative agricultural practices. They stress the importance of involving farmers in such research and in informing polices for implementation.

The UK is not the only country, of course, which faces the challenges of transforming their agrifood systems. The paper by De Matteis *et al.* [16] takes a global perspective of the problem, describing the challenges faced by all countries and emphasizing the importance of long-term programmatic and investment cycles that use the interconnections within the agrifood system and empower a diversity of voices to participate in decision making. Different countries must overcome different challenges and find different solutions. Some countries produce considerably more food than that required to meet its own needs, and yet food export driven by economic needs, results in dependence of food imports for food security. By contrast, as discussed by Li *et al.* [17], a country such as Singapore, a small island state with limited natural resources must import high volumes of food to meet its demand, so exposing itself to global market volatility. The article describes Singapore’s drive for innovation through a comprehensive framework based on technological innovation, regulatory agility and collaborative partnerships, aiming to serve as a benchmark for how to bring about a successful food system transformation.

So, what have we learned from the research carried out within the TUKFS programme, and how might its output be used to formulate the policies required for food system transformation in the UK? The concluding article by Bridle *et al.* [18] identifies the learning from TUKFS projects as a set of key suggested action areas for supporting food system transformation, across themes spanning production, manufacturing, supply chain and consumption, considering both community and policy, and with implications for practitioners and policy makers as well as researchers. The importance of co-creation in the design and involvement of all those involved in the food system from farmers to policy makers and community groups to large and small business during the research is highlighted as essential in the provision of evidence to inform decision makers on how to transform food systems to be healthier for people and the planet.

Yet, further barriers remain which prevent the translation of such evidence into practice, particularly in the policy space. Public sector decision makers are often unwilling to use coercive instruments owing to anticipated public backlash [19]. Since almost all agricultural and food policies are likely to redistribute economic advantages and disadvantages along the food supply chain, politicians charged with making such decisions are subject to lobbying and pressure from interest groups [20,21]. Policy choices thus consider both economic and political evidence in addition to scientific evidence. This may lead to the dilution or dismissal of key scientific evidence such as the need to take a systems approach to the agrifood sector [22]. This approach, which fully integrates food production and consumption with their impacts on health and environment, is the cornerstone of the research presented here and has been advocated by many agrifood researchers for over a decade. It is therefore welcome that the most recent food policy paper from the UK government department that oversees policy development in this field has specifically addressed actions within the *wider food system* and furthermore recommends setting up the Citizens Advisory Council and the Food Strategy Advisory Board [23]. Such stakeholder consultation and co-creation were also at the heart of many of the projects in the TUKFS programme and articles in this Theme Issue.

## Data Availability

This article has no additional data.
